# Correction: Microflower-like Co_9_S_8_@MoS_2_ heterostructure as an efficient bifunctional catalyst for overall water splitting

**DOI:** 10.1039/d2ra90081e

**Published:** 2022-08-30

**Authors:** Chaohai Pang, Xionghui Ma, Yuwei Wu, Shuhuai Li, Zhi Xu, Mingyue Wang, Xiaojing Zhu

**Affiliations:** Analysis and Test Center, Chinese Academy of Tropical Agricultural Sciences, Hainan Provincial Key Laboratory of Quality and Safety for Tropical Fruits and Vegetables, Key Laboratory of Quality and Safety Control for Subtropical Fruit and Vegetable, Ministry of Agriculture and Rural Affairs Haikou 571101 China 18389859589@163.com happylishuhuai@163.com; Key Laboratory of Tropical Fruits and Vegetables Quality and Safety for State Market Regulation Haikou 570311 China; Research Center of Advanced Chemical Equipment, Chemistry and Chemical Engineering Guangdong Laboratory Shantou 515041 China xiaoj_zhu@163.com

## Abstract

Correction for ‘Microflower-like Co_9_S_8_@MoS_2_ heterostructure as an efficient bifunctional catalyst for overall water splitting’ by Chaohai Pang *et al., RSC Adv.*, 2022, **12**, 22931–22938, https://doi.org/10.1039/D2RA04086G.

The authors regret that an incorrect grant number was shown in the acknowledgements section of the published article. The corrected section should read:

This work was financially supported by the Central Public-interest Scientific Institution Basal Research Fund (No.1630082022008). China Agriculture Research System of MOF and MARA (CARS-31). Key Laboratory of Tropical Fruits and Vegetables Quality and Safety for State Market Regulation (Grant no. ZX-2022002). Additional support was provided by the Foundation from Chemistry and Chemical Engineering Guangdong Laboratory (Grant No. 2111016) and the Basic and Applied Basic Research Foundation of Guangdong Province (Grant no. 2021A1515110111).

The Royal Society of Chemistry apologises for these errors and any consequent inconvenience to authors and readers.

## Supplementary Material

